# Enhanced Sugar and Bioethanol Production from Broom Grass via NaOH-Autoclave Pretreatment

**DOI:** 10.3390/polym17030266

**Published:** 2025-01-21

**Authors:** Duangporn Premjet, Siripong Premjet

**Affiliations:** 1 Department of Agricultural Science, Faculty of Agriculture, Natural Resources and Environment, Naresuan University, Phitsanulok 65000, Thailand; 2 Department of Biology, Faculty of Science, Naresuan University, Phitsanulok 65000, Thailand; 3 Center of Excellence in Research for Agricultural Biotechnology, Faculty of Agriculture, Natural Resources and Environment, Naresuan University, Phitsanulok 65000, Thailand

**Keywords:** broom grass, weed biomass, NaOH-autoclave pretreatment, glucose recovery, cellulosic ethanol

## Abstract

The effective utilization of nonfood biomass for bioethanol production represents a promising strategy for sustainable energy development. Moreover, limited research has been conducted on broom grass (*Thysanolaena latifolia*) as a potential feedstock for bioethanol production, particularly regarding the effects of NaOH autoclave pretreatment on its enzymatic digestibility and fermentability. This study optimized sodium hydroxide (NaOH) pretreatment combined with autoclaving to enhance the enzymatic digestibility of broom grass biomass. The effects of NaOH concentration (1–4%) and temperature (110–130 °C) on biomass composition, structural features, and enzymatic hydrolysis were systematically evaluated. Pretreatment with 2% NaOH at 120 °C emerged as optimal, achieving 74.7% lignin removal and 93.2% glucan recovery, thereby significantly improving enzymatic hydrolysis efficiency (88.0%) and glucose recovery (33.3%). Scanning electron microscopy (SEM) and X-ray diffraction (XRD) analyses revealed that these improvements were attributed to the increased surface porosity and the selective removal of amorphous components while maintaining cellulose crystallinity. The pretreated biomass hydrolysate exhibited excellent bioethanol production. Fermentation using *Saccharomyces cerevisiae* TISTR 5339 achieved an 86.4% ethanol conversion rate, yielding 147 g of bioethanol per 1000 g of pretreated biomass and representing a 2.6-fold increase compared to untreated feedstock. These findings demonstrate the potential of the NaOH autoclave pretreatment in enhancing bioethanol production from broom grass biomass, aiding the advancement of sustainable and cost-effective lignocellulosic biorefinery processes. The utilization of broom grass for bioethanol production presents an opportunity to valorize this multifaceted plant and expand its potential beyond its traditional uses.

## 1. Introduction

The advancement of efficient biorefinery technologies using sustainable plant materials is crucial to meet the growing global demand for biofuels and bioproducts. Lignocellulosic biorefineries present a promising approach for mitigating climate change and enhancing agricultural sustainability [1,2]. Lignocellulosic feedstocks, mainly comprising cellulose, hemicellulose, and lignin, form the primary structural components of plant cell walls and represent the most abundant source of renewable carbohydrates on Earth. Modern integrated biorefineries employ these materials to produce bioethanol and other high-value products [3].

Various lignocellulosic feedstocks have been investigated for bioethanol production, including agricultural residues (rice straw, corn stover, and wheat straw), woody materials (eucalyptus, pinewood, and bamboo) [1,4], and energy crops (switchgrass and miscanthus) [4,5]. Each feedstock type presents unique challenges and advantages. Agricultural residues offer abundance but face seasonal availability and collection logistics [6]. Woody materials provide year-round availability but require intensive pretreatment due to their rigid structure [7,8,9]. Energy crops offer high yields but compete for agricultural land [10,11]. In contrast, wild grass species like broom grass represent an attractive alternative due to their ability to grow on marginal lands, year-round availability, and minimal agricultural inputs [8,11].

Various weed species, such as *Sida acuta* [12], *Chloris barbata* [13], *Vietnamosasa pusilla* [14], *Lantana camara*, *Prosopis juliflora*, and *Saccharum spontaneum* [15] are being investigated as alternative energy sources owing to their high cellulose content and capacity to thrive in resource-limited environments. Our previous study demonstrated the high potential of broom grass (*Thysanolaena latifolia*), a prevalent species in northern Thailand, for bioethanol production [16]. In contrast to conventional food crops, broom grass is a non-edible tussock-forming grass that exhibits remarkable resilience under various environmental conditions, making it an ideal candidate for transforming underutilized wild species into valuable resources for rural economies [17].

The complex structure of lignocellulose poses challenges for its enzymatic degradation and bioenergy conversion [15]. Researchers have developed various pretreatment methods to overcome the natural resistance of lignocellulosic materials. Among pretreatment methods, physical (milling and irradiation), chemical (acid, alkaline, and oxidative), physicochemical (steam explosion and ammonia fiber explosion), and biological approaches have been studied extensively [1,18]. Sodium hydroxide (NaOH) pretreatment is a widely used chemical pretreatment method for converting lignocellulosic biomass into valuable products. This alkaline pretreatment effectively removes lignin and hemicellulose while partially solubilizing cellulose, thereby improving the biomass accessibility for further processing. This method not only enhances the efficiency of biofuel production but also promotes the overall sustainability of biorefineries [1,3,19].

Recent studies have explored combining NaOH pretreatment with different processing methods, such as autoclave- and microwave-assisted treatments, to enhance sugar yields [20]. The optimal combination of NaOH concentration and processing conditions is crucial for maximizing fermentable sugar production while maintaining process efficiency [21]. Various investigations have demonstrated that NaOH pretreatment combined with autoclaving markedly enhances enzymatic hydrolysis yields, as seen with elephant grass (314 mg/g glucose, 64 mg/g xylose) [22], rice straw (91.7% total sugar yield) [23], cassava peel (742.84 mg/g reducing sugar) [24], and wheat straw (75.2% enzymatic saccharification) [25]. However, limited research has explored broom grass as a feedstock for bioethanol production, particularly regarding the effects of NaOH autoclave pretreatment on its enzymatic digestibility and fermentability.

In this study, we aimed to investigate the potential of broom grass, an underutilized lignocellulosic biomass, as a feedstock for cellulosic ethanol production by evaluating the effectiveness of NaOH-autoclave pretreatment in improving its enzymatic digestibility and fermentability.

## 2. Materials and Methods

### 2.1. Materials

Component analysis of the broom grass biomass revealed various constituents on a dry weight basis. Structural carbohydrates included cellulose (32.9 ± 0.5% glucan) and hemicellulose (23.8 ± 0.2% xylan, 5.7 ± 0.0% arabinan) components. Lignin fractions included acid-soluble (5.9 ± 0.1%) and acid-insoluble (22.2 ± 0.5%) lignin, as demonstrated by Wongleang et al. [16].

For the fermentation process, the fermentation organism *Saccharomyces cerevisiae* TISTR 5339 was sourced from the Thailand Institute of Scientific and Technological Research (TISTR) in Khlong Luang, Thailand. The commercial enzyme used, *Trichoderma reesei* cellulase (Celluclast 1.5 L, product C2730), was sourced from Sigma-Aldrich, St. Louis, MO, USA. Almond-derived β-glucosidase (G0035) was procured from the Thailand facility of the Oriental Yeast Co., Ltd., Tokyo, Japan as described in a previous report [16].

### 2.2. Analytical Methods

Procedures for estimating cellulose, hemicellulose, and lignin [acid-soluble lignin (ASL) and acid-insoluble lignin (AIL)] quantities in raw and treated feedstocks followed the National Renewable Energy Laboratory guidelines [26]. Sugar and ethanol yields were determined as previously described [27]. In brief, an Agilent 110 high-performance liquid chromatography system (HPLC; Agilent Technologies, Santa Clara, CA, USA) equipped with an Aminex HPX-87P column (300 mm × 7.8 mm, Bio-Rad Laboratories, Hercules, CA, USA) and a refractive index (RI) detector (G1362A; Agilent Technologies) was used to determine the sugar and ethanol contents in the sample. HPLC-grade 0.005 M sulfuric acid was used in the mobile phase at a flow rate of 0.6 mL/min, with the column and RI detector maintained at 80 °C and 55 °C, respectively.

### 2.3. Pretreatment Procedure for Broom Grass Raw Material

The pretreatment procedure has been outlined previously [13]. To systematically optimize the pretreatment conditions, a two-stage approach was employed. First, the optimal NaOH concentration was determined by treating 3 g of raw material with different NaOH concentrations (1%, 2%, 3%, and 4% *w*/*v*) at a 1:8 ratio in 125 mL Erlenmeyer flasks, then wrapped in aluminum foil. The flasks were subsequently autoclaved at 120 °C (Hirayama, HVA-85, Toyono-cho, Kasukabe-shi, Saitama, Japan) under 15 psi for 1 h and allowed to cool to 25 °C. The mixture was transferred to 50 mL centrifuge bottles and centrifuged at 7000× *g* for 10 min to achieve component separation, after which the supernatant was discarded. The solid fraction was repeatedly washed with deionized water until a neutral pH was achieved. After determining the optimal NaOH concentration, the second optimization stage examined the effect of temperature by autoclaving the biomass at 110 °C, 120 °C, and 130 °C using the optimal NaOH concentration. Upon completion of the pretreatment, the solid fraction was extensively rinsed with deionized water to attain neutral pH levels. The optimization process used glucose recovery and enzymatic hydrolysis efficiency as key performance indicators.

Effectiveness was evaluated by quantifying lignin reduction and recovery yield and calculated using the following equations:

(1) $$Lignin\;reduction\left(\%\right)=100-lignin\;recovery$$

(2) $$Recovery\;yield\left(\%Dw\right)=SR\left(\%\right)\times treated\;component\;(\%Dw)/untreated\;component\left(\%Dw\right)$$

where SR and %Dw are solid recovery and percentage dry weight, respectively.

### 2.4. Crystallinity Analysis

Sample preparation for evaluating the crystallinity index (CrI) has been outlined in prior research [27]. In summary, treated and untreated biomass were subjected to three acetone rinses and subsequently air-dried at 25 °C. The dried samples were pulverized and screened through a 150 μm sieve. The crystalline structures of the samples were subsequently measured using X-ray diffraction (XRD; PANalytical X’pert Pro, PW 3040/60 diffractometer, Almelo, The Netherlands). The samples were scanned within a 2θ range from 10° to 40°, and the crystallinity of the material was quantified using the CrI as defined by Segal et al. [28]:

CrI (%) = [(I00_2_ − I_am_)/I00_2_] × 100 (3)

where I002 is the maximum intensity at 2θ = 22.0° (crystalline region), and I_am_ is the minimum intensity at 2θ = 15.9° (amorphous region).

### 2.5. Morphology Analysis

Sample preparation for microscopy using scanning electron visualization has been previously described [13]. In brief, untreated or NaOH-pretreated broom grass samples were freeze-dried and affixed to specimen stubs. Samples were sputter-coated with gold before visualization using field-emission scanning electron microscopy (SEM; LEO 1455VP, Zeiss, Gottingen, Germany).

### 2.6. Biomass Enzyme Saccharification of Broom Grass

The biomass saccharification process was performed according to Premjet et al. [27]. In brief, both treated and untreated broom grass biomass were evaluated for dry weight using a maximum of 10 mL in a 50 mL shaking container at 50 °C and 150 rpm for 96 h (Innova 4340, New Brunswick Scientific Company, Edison, NJ, USA). The enzyme hydrolysis reaction comprised 0.1 g of dry biomass, 0.05 mol/L of sodium citrate buffer (pH 4.8), 0.1 mL of 2% sodium azide (*w*/*v*), 30 FPU/g of cellulase (Celluclast 1.5 L), and 60 U/g of β-glucosidase (dry biomass). Hydrolysates were collected at 12, 24, 48, 72, and 96 h to quantify glucose concentrations via HPLC. The hydrolysis efficiency of glucan (HEG) and glucose recovery (GR) were assessed using Equations (4) and (5), respectively:

(4) $$HEG=\frac{glucose\;release\;(g)\times 0.9\times 100}{initial\;glucan\;biomass\;(g)}$$

(5) $$GR\left(\%\right)=SR\left(\%\right)\times glucan\left(\%\right)\times 1.11\times HEG\left(\%\right)\times 100$$

where SR is the solid recovery, and 0.9 and 1.11 represent the conversion coefficients of cellulose to glucose.

### 2.7. Biomass Hydrolysate Preparation

Biomass hydrolysate was prepared according to the methodology outlined by Premjet et al. [12]. Briefly, the biomass hydrolysate of broom grass was generated via enzyme saccharification of the optimally pretreated biomass (2% NaOH at 120 °C for 60 min) and subsequently treated at 100 °C for 20 min in a water bath. Subsequently, the biomass hydrolysate was centrifuged at 12,000× *g* and 10 °C for 2 h. The supernatant was filtered through a glass filter. The solution was subsequently evaporated using a rotary evaporator (Heidolph Instruments, Hei-VAP Advantage, GmbH & Co. KG Walpersdorfer Str., Schwabach, Germany) to achieve a glucose concentration of approximately 20 g/L, after which 1 M NaOH was added to increase the pH to 6. The biomass hydrolysate was subsequently stored at 4 °C for future experiments, remaining undetoxified.

### 2.8. Ethanol Fermentation Medium

Ethanol fermentation and yield were assessed as outlined in Premjet et al. [12]. A total of two types of fermentation media were prepared: biomass hydrolysate medium (BHM) containing 20 g/L glucose from pretreated biomass (described in Section 2.6), and synthetic glucose medium (SGM) using 20 g/L analytical quality commercial glucose (98%) as a control. Both media were supplemented with identical nutrients (10 g/L yeast extract, 10 g/L peptone) and minerals (2 g/L K_2_HPO_4_, 2 g/L MgSO_4_), with pH adjusted to 6.0. The media were sterilized using a 0.02-µm Millipore filter before inoculation with 2% (*v*/*v*) yeast. Fermentation was performed at 30 °C in a shaking incubator (Innova 4340; New Brunswick Scientific) at 150 rpm for 24 h, with solution components collected at 3, 6, 9, 12, 15, 18, 21, and 24 h for HPLC analysis of sugar consumption and ethanol production.

Ethanol yield % was calculated utilizing Equation (6):

(6) $$Ethanol\;yield\left(\%\right)=\frac{Amount\;of\;ethanol\;generated\left(g\right)\times 100}{0.511\times amount\;of\;initial\;glucose\;(g)}$$

### 2.9. Culture Preparation

Culture preparation and yeast cell proliferation followed the methods outlined by Premjet et al. [12]. In short, yeast from the agar slant was inoculated into 10 mL of yeast malt (YM) broth and cultivated at 30 °C for 18 h in a rotating-shaker chamber set at 180 rpm. The proliferation of yeast cells at an optical density (OD) wavelength of 600 nm was measured using ultraviolet (UV) spectrophotometry (SP-830 Plus; Metertech, Taipei, Taiwan). An OD at 600 nm equivalent to one corresponded to approximately 1.5 × 10^7^ cells/mL.

### 2.10. Statistical Analysis

In this study, an analysis of variance (ANOVA) was performed utilizing IBM SPSS software (SPSS 26.0 for Windows; SPSS Inc., Chicago, IL, USA). Each test was performed at least three times, and the final results were expressed as mean values. The differences between mean values were determined using Duncan’s multiple-range test (DMRT) at a significance level of 95% (*p* < 0.05).

## 3. Results

### 3.1. Effects of NaOH Concentration Pretreatment on Biomass Fractionation

The impact of NaOH concentration combined with autoclaving (120 °C, 15 psi, 1 h) on the biomass composition and CrI of broom grass biomass is presented in Table 1. As NaOH concentrations increased from 1% to 4%, xylan content significantly decreased from 19.8 ± 0.3% with 1% NaOH to 15.4 ± 0.2% with 4% NaOH, whereas arabinan decreased from 4.9 ± 0.2% to 3.8 ± 0.0% within the same concentration range. Compared to the control sample, the treated sample exhibited a 16.8–35.3% decrease in xylan and a 14.0–33.3% decrease in arabinan. The most pronounced effect was observed for lignin removal. The total lignin content decreased substantially from 28.2 ± 0.4% in the untreated biomass to 16.1 ± 0.4% with 1% NaOH and further to 10.9 ± 0.1% with 4% NaOH, corresponding to lignin reductions of 42.9% and 61.3%, respectively. Both acid-insoluble lignin (AIL) and acid-soluble lignin (ASL) exhibited significant reductions, with AIL decreasing from 22.2 ± 0.5% to 8.6 ± 0.1% and ASL decreasing from 5.9 ± 0.1% to 2.4 ± 0.0% with 4% NaOH. Glucan content increased significantly from 48.3 ± 0.2% with 1% NaOH to 61.3 ± 0.3% with 4% NaOH (*p* < 0.05), representing increases of 46.8% and 86.3% compared to the untreated biomass (32.9 ± 0.5%), respectively. In contrast, hemicellulose components exhibited a decreasing trend.

**Figure 1:**
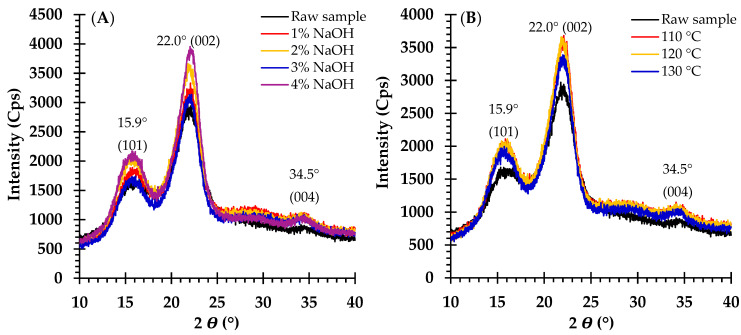
X-ray diffractograms of broom grass samples: (**A**) raw and NaOH-pretreated (1–4%) biomass, and (**B**) raw and temperature-treated (110–130 °C) biomass with 2 % NaOH-autoclave pretreatment.

The X-ray diffractogram pattern indicated that both the untreated and treated materials displayed two distinct peaks at 2θ angles of approximately 15.9° and 22.2°, consistent with cellulose I, as depicted in Figure 1. The untreated sample had a CrI of 54.1%. Increasing the NaOH concentration from 1% to 4% increased the CrI from 57.8% to 65.1% (Table 1 and Figure 1A).

Table 1 presents the recovery yields of the various biomass components after NaOH pretreatment combined with autoclaving. The solid recovery yield decreased with increasing NaOH concentration, declining from 62.5 ± 0.5% with 1% NaOH to 48.9 ± 0.5% with 4% NaOH, indicating increased solubilization of biomass components at elevated alkali concentrations. Glucan recovery remained relatively stable across all NaOH concentrations, ranging from 91.1 ± 0.5% to 93.2 ± 0.3%, with peak recovery observed with 2% NaOH. This suggested that the cellulose fraction was predominantly preserved during pretreatment. Xylan and arabinan recovery yields consistently decreased with increasing NaOH concentrations. Xylan recovery decreased from 52.0 ± 0.7% with 1% NaOH to 31.7 ± 0.5% with 4% NaOH, while arabinan recovery decreased from 53.9 ± 2.3% to 32.1 ± 0.3% over the same range. The most significant impact was observed in lignin removal, which increased from 64.2 ± 1.0% with 1% NaOH to 81.0 ± 0.1% with 4% NaOH, corresponding to a decrease in total lignin recovery from 35.8 ± 1.0% to 19.0 ± 0.1%.

**Figure 2:**
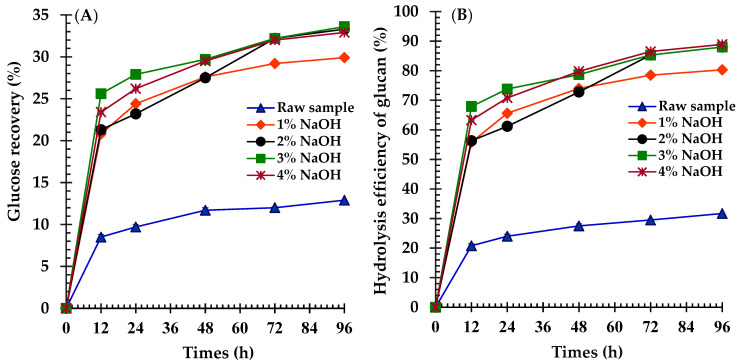
Enzymatic hydrolysis profiles of raw and NaOH-pretreated (1–4%) broom grass showing: (**A**) glucose recovery (GR), and (**B**) hydrolysis efficiency of glucose (HEG) over 96 h of incubation.

The effect of NaOH pretreatment combined with autoclaving on the enzymatic digestibility of broom grass biomass was evaluated via GR and the HEG yields over 96 h (Figure 2A,B). Untreated biomass exhibited low GR and HEG yields (12.9 ± 0.1% and 31.7 ± 0.3% at 96 h, respectively). NaOH pretreatment significantly enhanced both metrics at all tested concentrations (*p* < 0.05). The 1% NaOH pretreatment more than doubled the GR yield to 29.9 ± 0.1% and increased the HEG yield to 80.3 ± 0.2% at 96 h. Higher NaOH concentrations (2%, 3%, and 4%) further improved performance, yielding statistically similar 96 h GR yields (33.3 ± 0.2%, 33.3 ± 0.1%, and 32.9 ± 0.2%, respectively) and HEG yields (88.0 ± 0.4%, 88.0 ± 0.1%, and 88.9 ± 0.6%, respectively), with no significant differences among them (*p* > 0.05). Notably, the pretreated samples demonstrated rapid initial rates for both the GR and HEG yields, contrasting with the gradual increase observed in the untreated samples.

### 3.2. Effects of Temperature on Biomass Fractionation

To determine the optimal temperature for pretreating broom grass biomass, the feedstock was treated with 2% NaOH in an autoclave at 110 °C, 120 °C, and 130 °C to evaluate the effect of temperature on its chemical structure and CrI (Table 2). Increasing the pretreatment temperature from 110 °C to 130 °C resulted in significant decreases in the AIL, ASL, and total lignin content (Table 2). The AIL decreased most from 14.9 ± 0.3% at 110 °C to 8.5 ± 0.4% at 130 °C. The ASL decreased from 2.9 ± 0.1% to 2.4 ± 0.1%, while the total lignin content decreased from 17.9 ± 0.3% to 10.9 ± 0.4%. The lowest lignin content was observed at 130 °C. Conversely, glucan content increased from 49.7 ± 0.6% to 57.1 ± 0.2%. Xylan and arabinan exhibited minor decreases, with xylan decreasing from 20.8 ± 0.4% to 18.7 ± 0.1% and arabinan decreasing from 4.8 ± 0.1% to 4.5 ± 0.0% across the same temperature range.

After pretreating the raw material with 2% NaOH in an autoclave at different temperatures, the XRD patterns revealed that both the untreated and treated materials maintained the two distinct peaks of cellulose I, as observed in Section 3.2. Maintaining a 2% NaOH concentration and increasing the temperature from 110 °C to 130 °C slightly increased the CrI from 60.7% to 62.2% (Table 2 and Figure 1B).

Table 2 shows the recovery yields of biomass components after pretreatment at various temperatures. Solid recovery decreased from 58.2 ± 0.5% at 110 °C to 54.2 ± 0.6% at 130 °C. Glucan recovery increased from 87.9 ± 1.0% at 110 °C to 93.2 ± 0.4% at 120 °C, remaining statistically similar with 94.0 ± 0.2% at 130 °C (*p* > 0.05). In contrast, increasing the temperature decreased the recoveries of xylan, arabinan, AIL, ASL, and the total lignin. Xylan recovery decreased from 50.8 ± 0.9% at 110 °C to 42.6 ± 0.2% at 130 °C, while arabinan decreased from 48.4 ± 0.7% to 42.7 ± 0.3%. The AIL, the ASL, and the total lignin recoveries decreased from 29.1 ± 0.7%, 28.8 ± 0.9%, and 36.9 ± 0.6% at 110 °C to 20.7 ± 1.1%, 21.9 ± 0.6%, and 20.9 ± 0.8% at 130 °C, respectively. The lignin removal increased progressively with temperature, rising from 63.1 ± 0.3% at 110 °C to 74.7 ± 0.1% at 120 °C and 79.1 ± 0.8% at 130 °C. It achieved the highest decrease at 130 °C.

**Figure 3:**
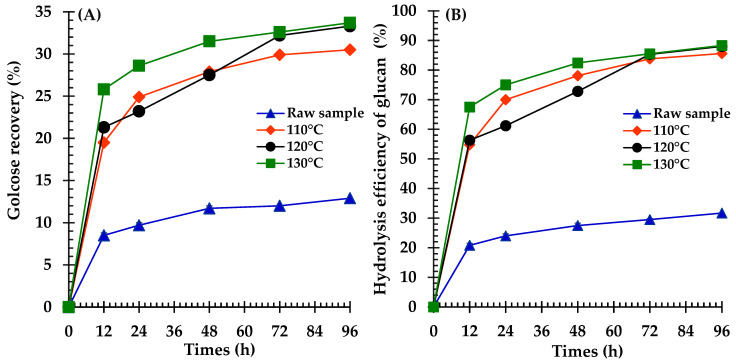
Enzymatic hydrolysis profiles of raw and temperature-treated (110–130 °C) broom grass with 2% NaOH showing: (**A**) glucose recovery (GR), and (**B**) hydrolysis efficiency of glucose (HEG) over 96 h of incubation.

Both the untreated and treated broom grass samples were subjected to enzymatic hydrolysis to determine the optimal pretreatment temperature (Figure 3A,B). After 96 h of hydrolysis, the GR and HEG yields of the treated and untreated biomasses reached their maximum levels. The treated feedstocks exhibited higher GR and HEG yields than the untreated materials. After treatment at 110, 120, and 130 °C, the GR rates were 30.5 ± 0.5%, 33.3 ± 0.2%, and 33.7 ± 0.1%, whereas the HEG yields were 85.6 ± 1.4%, 88.0 ± 0.4%, and 88.3 ± 0.2%, respectively. Notably, no significant variations were observed in the GR and HE yields between 120 °C and 130 °C. However, the untreated sample yielded the lowest GR (12.9 ± 0.1%) and HEG (31.7 ± 0.3%).

### 3.3. Effects of NaOH and Temperature Pretreatment on Morphological Structure

**Figure 4:**
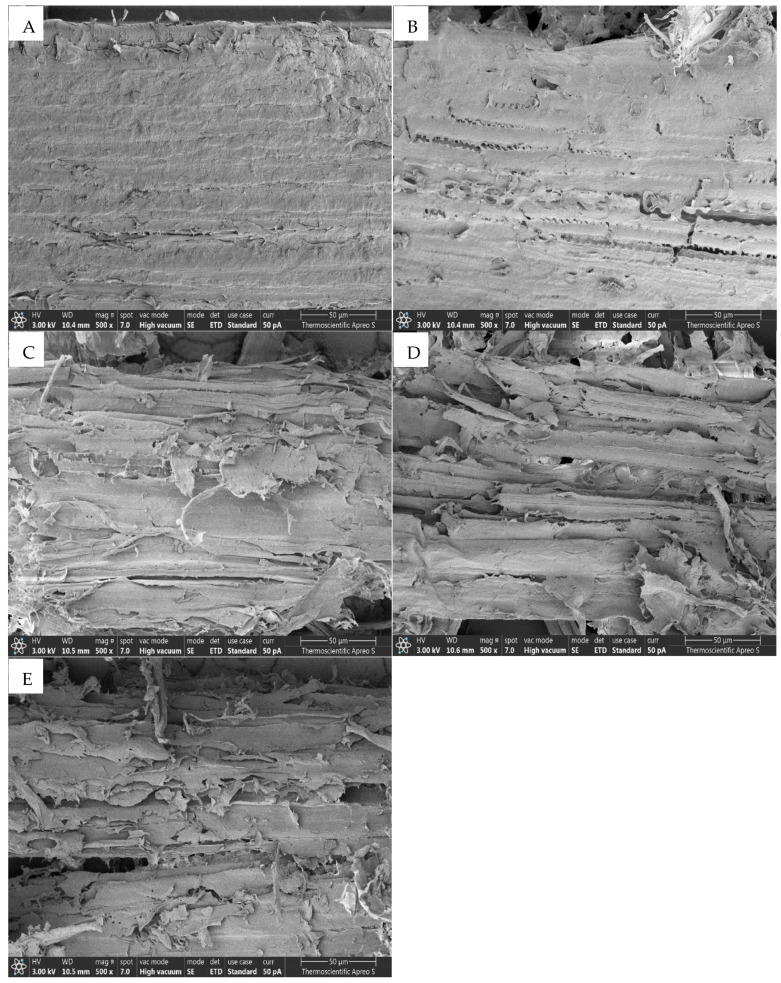
SEM micrographs (500×) showing the surface morphology of broom grass: (**A**) untreated biomass, and biomass pretreated with (**B**) 1%, (**C**) 2%, (**D**) 3%, and (**E**) 4% NaOH at 120 °C.

**Figure 5:**
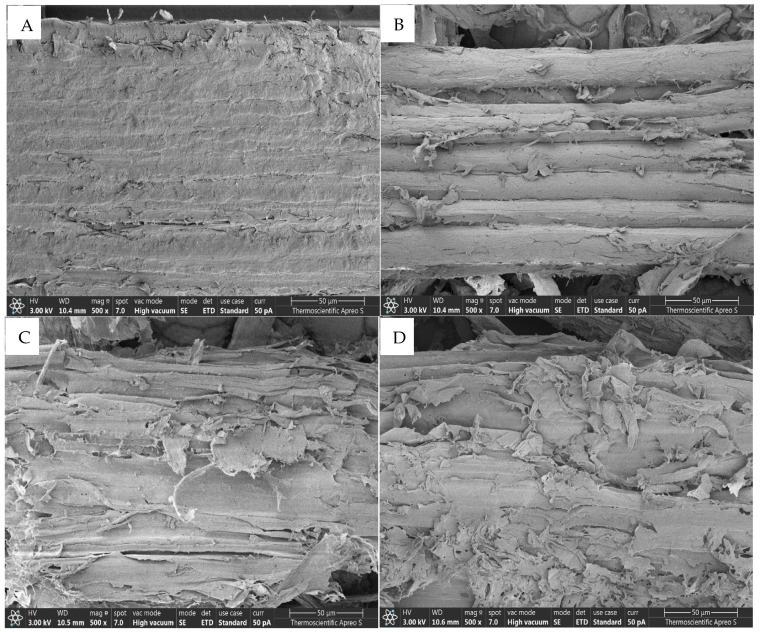
SEM micrographs (500×) showing the surface morphology of broom grass: (**A**) untreated biomass, and biomass pretreated with 2% NaOH at (**B**) 110 °C, (**C**) 120 °C, and (**D**) 130 °C.

We employed SEM to examine the morphological changes in treated and untreated broom grass material. Treating broom grass biomass at 120 °C with increasing NaOH concentrations and different temperatures resulted in progressive changes in surface morphology (Figure 4A–E and Figure 5A–D). The untreated biomass featured a smooth and compact surface that gradually evolved as the NaOH concentration increased (Figure 4A). Minor surface changes appeared with 1% NaOH (Figure 4B), whereas treatment with 2% NaOH resulted in increased roughness and porosity (Figure 4C). Subsequently, with 3% NaOH, further alterations in surface irregularities transpired, culminating in considerable modifications, visible fibrillation (Figure 4D), and increased porosity with 4% NaOH concentration (Figure 4E). Furthermore, surface changes occurred with 2% NaOH treatment at 110 °C, exhibiting slight roughening and initial signs of fiber separation (Figure 5B). However, at 130 °C, significant structural changes were observed, characterized by extensive fiber separation, increased surface area, and pronounced porosity (Figure 5D).

### 3.4. Ethanol Fermentation

**Figure 6:**
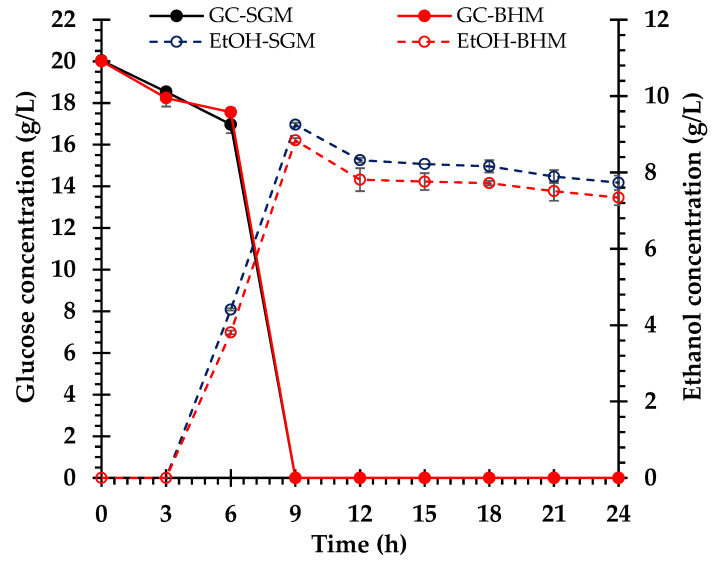
Time-course profiles of glucose consumption (GC) and ethanol production (EtOH) during fermentation by *S. cerevisiae* TISTR 5339 in the synthetic glucose medium (SGM) and the biomass hydrolysate medium (BHM).

**Figure 7:**
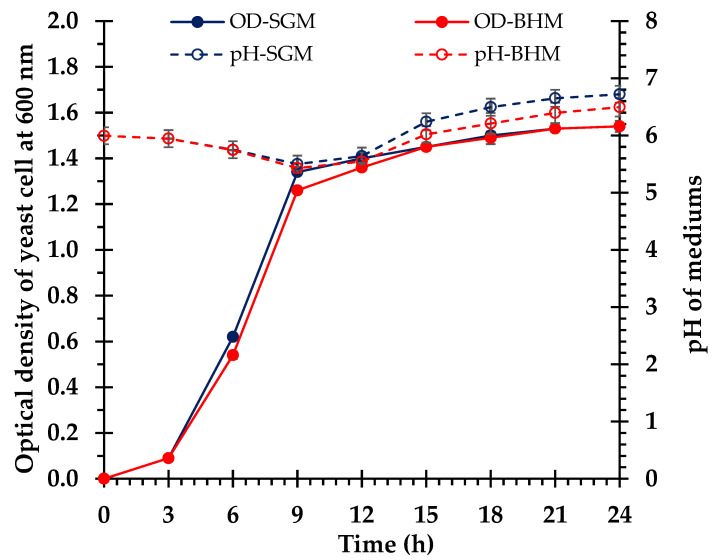
Growth characteristics of *S. cerevisiae* TISTR 5339 showing the cell density (OD) and the pH changes during fermentation in the synthetic glucose medium (SGM) and the biomass hydrolysate medium (BHM).

In this study, *S. cerevisiae* TISTR 5339 was used for ethanol fermentation in both the SGM and the non-detoxified BHM. We compared the fermentation and growth of *S. cerevisiae* TISTR 5339 in the SGM and BHM. Both media commenced with 20.0 g/L of glucose, which was completely consumed within 9 h, indicating efficient glucose utilization. The SGM demonstrated accelerated initial glucose uptake (0–3 h), whereas the BHM showed higher consumption (3–6 h). Ethanol production in both media commenced between 3 and 6 h. By 9 h, the SGM peaked at 90.9 ± 0.8% or 9.3 ± 0.0 g/L, slightly higher than the BHM at 86.4 ± 0.0% or 8.8 ± 0.1 g/L, thereby indicating a greater potential for ethanol yield in the SGM. Both media then experienced decreases in ethanol concentrations to approximately 75.6 ± 0.7% or 7.7 ± 0.2 g/L in the SGM and 71.4 ± 1.4% or 7.3 ± 0.2 g/L in the BHM from 9 to 24 h (Figure 6). Growth patterns were similar, with the OD increasing to 1.5 and stabilizing at 15 h. The pH levels varied among the media owing to their compositions, with the SGM showing greater pH variation than the BHM (Figure 7).

## 4. Discussion

### 4.1. Effects of NaOH Concentration and Temperature on Biomass Fractionation

The significant changes in chemical composition and component recovery yields illustrate the effective fractionation of broom grass biomass via alkaline pretreatment. NaOH concentration and temperature are pivotal factors in this process. The progressive increases in glucan content (from 32.9% to 61.3% with increasing NaOH and from 49.7% to 57.1% with increasing temperature) can be attributed to the preferential removal of non-cellulosic components, particularly lignin and hemicelluloses, resulting in the enrichment of the cellulosic fraction in the pretreated solids [23,29,30,31,32,33,34].

The substantial decreases in lignin contents achieved through both the increased NaOH concentration (28.2% to 10.9%) and the increased temperature (17.9% to 10.9%) indicate effective delignification, achieving up to 81% removal with 4% NaOH and 79.1% removal at 130 °C. These phenomena can be explained by the synergistic effects of alkaline and thermal treatments, aligning with previous research findings [13,31,33,35]. The mechanism of lignin removal during NaOH-autoclave pretreatment follows a sequential process: (1) NaOH initially breaks the ester bonds between lignin and hemicellulose through saponification [36,37]. This step disrupts the lignin–carbohydrate complexes (LCC) that link lignin to hemicellulose in the plant cell wall [38]. (2) The elevated temperature and pressure from autoclaving then facilitate the cleavage of both α-aryl and β-aryl ether bonds within the lignin structure [36,37]. These ether bonds are the primary linkages holding the lignin polymer together. (3) This combined effect leads to lignin fragmentation and solubilization [36,37,39]. As the lignin structure is broken down, it becomes more soluble in the alkaline solution and can be removed from the biomass. Moreover, differential removal rates exist between the AIL and the ASL, with the ASL, owing to its lower molecular weight [40], typically exhibiting greater susceptibility to removal under pretreatment conditions. Delignification is crucial for improving biomass digestibility [22,25].

The decreases in the hemicellulose components (xylan and arabinan) with increasing NaOH concentration and temperature indicate significant solubilization of these polysaccharides. The recovery yields indicate that xylan and arabinan recoveries decreased to approximately 32% with 4% NaOH and 42% at 130 °C. The removal of hemicelluloses can be attributed to the combined effects of alkaline deacetylation of xylan, the cleavage of ester linkages between hemicelluloses and other cell wall components [38,41], and the accelerated hydrolysis of glycosidic bonds at elevated temperatures [5,42,43,44].

The recovery efficiencies of solids, xylan, arabinan, glucan, and lignin sharply decreased with increasing NaOH concentrations and temperatures (Table 1 and Table 2), resulting in an increased degradation of both carbohydrate and non-carbohydrate components in the feedstock [45,46]. Increasing the NaOH concentration maintained glucan recoveries at between 91% and 93% (Table 1), whereas higher temperatures boosted recovery to 94% at 130 °C (Table 2). This suggests that the cellulose fraction was largely preserved during pretreatment. When comparing the primary carbohydrate components of broom grass feedstock, glucan was less susceptible to NaOH and temperature degradation than xylan because of the glucose being present in the crystalline cellulose form. Conversely, xylan and arabinan were present in the amorphous hemicellulose form [47]. This finding can be attributed to the synergistic effects of NaOH concentration and temperature, which significantly influenced the fractionation of broom grass biomass. Solid recovery diminished substantially because both pretreatment procedures accelerated the breakdown of certain amorphous components, such as lignin and hemicellulose [22]. The quantity of sugar recovered is greatly affected by the loss of solids during the pretreatment process, particularly cellulose, which is an essential cause of concern [13].

### 4.2. Effects of NaOH Concentration and Temperature on Crystallinity

The alkali pretreatment of lignocellulosic materials enhances biomass disintegration and alters cellulose crystallinity [4]. This study revealed that the CrI increased with both the NaOH concentration (from 54.1% to 65.1%) and the temperature (from 60.7% to 62.2%). These results indicate that increasing the NaOH concentration and temperature during autoclaving improves the effectiveness of the alkaline treatment, likely by facilitating NaOH penetration into the biomass structure. These results are consistent with those of previous studies [22,30,48,49]. Despite the increased CrI values in all treated samples, the XRD pattern consistently corresponded to that of cellulose I (Figure 1A,B). The mild alkaline conditions (1–4% NaOH) and relatively low temperatures (110–130 °C) are insufficient to induce the transformation of cellulose I into cellulose II [18]. Under all treatment conditions, the CrI consistently increased, with the NaOH concentration showing a more pronounced effect than the temperature. However, a higher CrI does not inhibit the removal of hemicellulose and lignin [50]. This modification indicates that higher NaOH concentrations and temperatures are more effective at removing hemicellulose and other amorphous components, thereby increasing the relative crystallinity of the treated samples [5,51,52].

### 4.3. Effects of the NaOH Concentration and the Temperature on the Morphological Structure

SEM analysis revealed that different NaOH concentrations and pretreatment temperatures caused significant morphological changes on the surface of the broom grass biomass, disrupting the compact, ordered structure while increasing porosity and surface area. These physical modifications can be attributed to the combined chemical effects of NaOH and temperature in disrupting the lignocellulosic matrix and the influence of pretreatment severity on the extent of structural disruption (Figure 4 and Figure 5). The smooth, intact surface of the untreated biomass reflects the presence of lignin, which serves as a structural sealant that binds the cellulose fibers together. Lignin is a hydrophobic aromatic polymer that occupies the spaces between cellulose and hemicellulose, provides mechanical strength, and restricts enzyme access to polysaccharides [3,15]. The lack of visible pores in the untreated biomass indicates the limited accessibility of hydrolytic enzymes to cellulose.

NaOH pretreatment disrupts the lignin–carbohydrate complex by cleaving ester bonds that cross-link lignin to hemicellulose, hydrolyze glycosidic linkages in hemicellulose, and solubilize lignin fragments [33,34,50,53]. This results in the separation and exposure of cellulose fibrils, the creation of pores, and the general loosening of the biomass structure, as evidenced by the SEM images. SEM analysis of pretreated broom grass biomass via both pretreatment processes elucidates the critical structure–function relationships that underpin the enhanced enzymatic hydrolysis efficiency. The development of porous structures and the progressive breakdown of rigid biomass architecture are directly correlated with increased enzyme accessibility. Consequently, structural modifications significantly affect enzymatic hydrolysis outcomes [13,31,33,54,55].

### 4.4. Effects of NaOH Concentration and Temperature on Glucose Recovery Yields

The substantial improvements in GR and HEG from broom grass biomass pretreated with NaOH at various concentrations (1–4%) and temperatures (110–130 °C) compared to untreated biomass illustrate the effectiveness of alkaline pretreatment in enhancing enzymatic saccharification. NaOH pretreatment likely increases glucose yield by modifying the chemical composition and physical structure of the biomass, thereby improving cellulose accessibility to enzymes (Figure 2 and Figure 3).

Increasing the NaOH concentration from 1% to 4% at 120 °C resulted in greater delignification (up to 81% removal) and hemicellulose solubilization, as indicated by the decreases in lignin, xylan, and arabinan contents in the pretreated solids. The enhanced enzymatic hydrolysis efficiency can be directly linked to the mechanism of lignin removal, as described in Section 4.1. As lignin is progressively removed through ester bond cleavage and fragmentation, three key factors contribute to improved enzyme accessibility: (1) increased surface porosity from the removal of lignin’s physical barrier [56,57], (2) reduced non-productive binding of enzymes to lignin surfaces [58], and (3) enhanced cellulose fiber swelling due to the disruption of the lignin–carbohydrate matrix [56,59]. The strong correlation between lignin removal (up to 81%) and hydrolysis efficiency (up to 88.0%) demonstrates the crucial role of comprehensive delignification in enabling effective enzymatic action. Hemicellulose can also hinder cellulose accessibility and nonspecifically bind cellulases [60]. Therefore, the decrease in lignin and hemicellulose owing to increased NaOH concentration accounts for the enhanced GR and HEG yields.

Increasing the pretreatment temperature from 110 °C to 130 °C with 2% NaOH also enhanced the GR and HEG yields, likely due to the accelerated delignification and hemicellulose removal at higher temperatures [42,43]. This can cause a greater disruption of lignin–carbohydrate complexes, an increased swelling of cellulose fibers, and alterations in cellulose crystallinity and polymerization degree, all of which facilitate enzymatic deconstruction [42,61].

The results revealed that higher relative CrI values in the treated samples from both pretreatment processes did not affect the enzymatic hydrolysis yield of the biomass. This phenomenon, noted by several authors, is attributed to enhanced delignification resulting from alkaline pretreatment, which increases cellulose accessibility [5,25]. Moreover, lignin is the primary inhibitor of cellulose enzymatic hydrolysis because it acts as a steric barrier to cellulase, preventing it from accessing the cellulosic substrate and reducing enzyme activity because of nonproductive absorption [62,63]. Therefore, these parameters have a greater impact on improving enzymatic hydrolysis efficiency, regardless of higher CrI values [25,43]. This finding is valuable for applications requiring both high digestibility and the maintenance of cellulose structural properties, such as bioethanol production or biomaterial development.

The current study found no significant difference (*p* > 0.05) in the GR and HEG yields from broom grass treated with 2–4% NaOH at 120 °C compared to those treated with 2% NaOH at 120–130 °C. Therefore, the optimal condition for achieving the highest GR (33.3 ± 0.2%) and HEG (88.0 ± 0.4%) yields was 2% NaOH at 120 °C for 60 min, resulting in approximately 75% delignification. The GR and HEG levels were approximately 2.6 and 2.8 times higher, respectively, than those of the raw sample. Furthermore, compared to other studies using NaOH pretreatment for various biomass sources, our results show a comparable or improved performance. Table 3 presents a comparative assessment of alkaline pretreatment conditions and results across various lignocellulosic substrates. Under similar pretreatment conditions (2% NaOH, 110–120 °C, 60 min), different biomass sources showed varying responses. For instance, *Chloris barbata* achieved 71% lignin removal with 30.7% glucose recovery [13], while Durian peel varieties showed a slightly higher performance (74–77% lignin removal, 34.9–36.1% glucose recovery) [31]. *Sicyos angulatus*, despite a similar NaOH concentration, showed lower glucose recovery (8.5%), even with a shorter treatment time [32]. Although *Vietnamosasa pusilla* demonstrated a higher glucose recovery (42.4%), it showed a lower lignin removal (50.5%) [33]. These comparisons indicate that broom grass, achieving 74.7% lignin removal and 33.3% glucose recovery under our optimized conditions, responds effectively to NaOH pretreatment and represents a promising feedstock for bioethanol production.

### 4.5. Comparative Fermentation Efficiency of S. cerevisiae in the Control and Biomass Hydrolysate Media

The fermentation characteristics of *S. cerevisiae* TISTR 5339 in the SGM and BHM demonstrated efficient ethanol generation from broom grass biomass. Similar trends in glucose consumption, ethanol production, cell growth, and pH changes in both media indicate that the BHM facilitated yeast fermentation similarly to the SGM. The rapid and complete depletion of glucose within 9 h in both media demonstrates that *S. cerevisiae* efficiently utilizes available sugars for growth and ethanol production. The slightly lower glucose concentrations in the BHM than in the SGM at 3 and 6 h (Figure 5) may be ascribed to the presence of other sugars (e.g., xylose and arabinose) in the hydrolysate that are not fully fermentable by the wild-type yeast strain, resulting in a diauxic shift during glucose and xylose cocultures [64,65]. Nonetheless, the complete consumption of glucose in the BHM demonstrates that the hydrolysate contained sufficient fermentable sugars derived from the enzymatic saccharification of broom grass biomass.

The ethanol yields in both media were comparable, with maximum concentrations of 90.9 ± 0.8% or 9.3 ± 0.0 g/L in the SGM and 86.4 ± 0.0% or 8.8 ± 0.1 g/L in the BHM at 9 h, corresponding to ethanol yields of 0.47 g/g and 0.44 g/g glucose, respectively. These yields approach the theoretical maximum of 0.51 g ethanol g/glucose, signifying the efficient conversion of sugars to ethanol by *S. cerevisiae* [66]. The slightly lower ethanol yield in the BHM may result from the allocation of sugars for yeast biomass production or the presence of minor inhibitory compounds in the hydrolysate [64,67]. When lignocellulosic biomass is subjected to high temperature and pressure, it produces various inhibitors, such as phenolic substances, furan derivatives, and weak acids [68,69]. The present study indicates that the BHM did not undergo detoxification, suggesting the potential presence of trace inhibitors. Previous studies have reported that furan derivatives have a more pronounced effect on inhibiting cell proliferation but have no substantial impact on ethanol production by *S. cerevisiae* [67,68,69]. This finding aligns with those of previous studies that reported similar outcomes when using alkaline pretreatment methods [12,33].

The decrease in ethanol concentration after 9 h in both media may be attributed to ethanol evaporation during sampling [70] or its consumption by yeast as a carbon source under glucose-depleted conditions [71]. However, ethanol levels remained relatively stable between 9 and 24 h, indicating that *S. cerevisiae* has high ethanol tolerance and can maintain its viability and metabolic function in the presence of ethanol [72].

**Figure 8:**
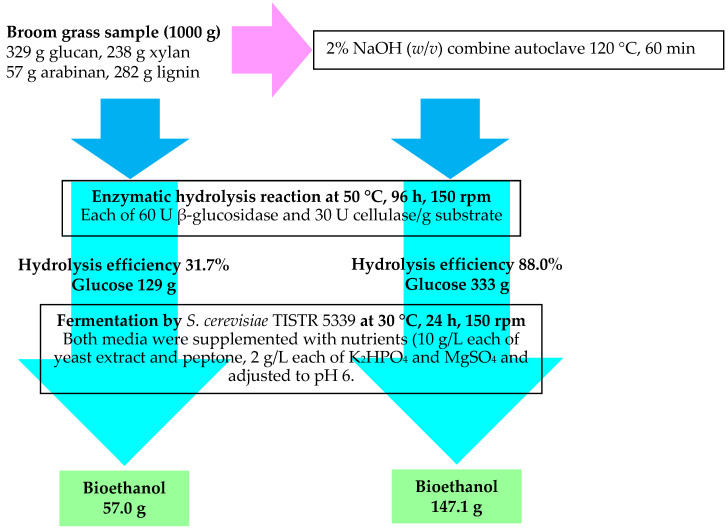
Process flow diagram and mass balance for bioethanol production from broom grass biomass (1000 g initial dry weight) showing the pretreatment (2% NaOH, 120 °C), enzymatic hydrolysis (50 °C, 96 h), and fermentation steps.

The mass balance analysis of bioethanol production from broom grass biomass revealed the significant impact of NaOH pretreatment on improving overall process efficiency (Figure 8). The pretreatment of 1000 g of broom grass biomass with 2% NaOH at 120 °C for 60 min resulted in a solid recovery of 56.2% (562 g), which was enriched in glucan (307 g) and exhibited lower xylan (109 g), arabinan (26 g), and lignin (71 g) contents compared to the initial biomass. The pretreated biomass achieved a remarkably high enzymatic hydrolysis efficiency of 88.0%, yielding 333 g of glucose, whereas the untreated biomass showed a hydrolysis efficiency of only 31.7%, yielding 129 g of glucose. The fermentation of glucose-rich hydrolysates with *S. cerevisiae* TISTR 5339 produced an ethanol yield of 86.4% for both untreated and pretreated biomass. However, the actual ethanol production from the pretreated biomass (147.1 g) was 2.6 times higher than that from the untreated biomass (57.0 g), attributable to the enhanced glucose release during hydrolysis. The overall process output of bioethanol from pretreated broom grass biomass was 14.7%, representing a substantial improvement over the 5.7% yield obtained from untreated biomass.

These results underscore the critical role of effective pretreatment in overcoming the recalcitrance of lignocellulosic biomass and maximizing the conversion of carbohydrates to fermentable sugars and, ultimately, bioethanol. The mass balance analysis provides valuable insights into the efficiency of each processing step and highlights the potential of broom grass as a promising feedstock for sustainable bioethanol production when combined with optimized pretreatment, hydrolysis, and fermentation strategies.

## 5. Conclusions

In this study, we comprehensively investigated broom grass (*Thysanolaena latifolia*), an abundant non-food biomass in northern Thailand, as a viable feedstock for bioethanol production. The optimized NaOH-autoclave pretreatment (2% NaOH, 120 °C, 60 min) considerably improved biomass digestibility, achieving 74.7% lignin removal and maintaining 93.2% glucan recovery. This pretreatment strategy led to a significantly enhanced enzymatic hydrolysis efficiency (88.0%) and GR (33.3%), representing a 2.8-fold increase relative to the untreated biomass. Notably, this study reveals that high digestibility can be achieved while maintaining cellulose crystallinity, providing new insights into structure–function relationships in biomass conversion. Additionally, the process exhibits excellent fermentation efficiency using *S. cerevisiae* TISTR 5339, achieving an 86.4% ethanol conversion rate without requiring detoxification steps. The optimized process yielded 147 g of bioethanol per 1000 g of the pretreated biomass, representing a 2.6-fold increase compared to the untreated biomass. These findings validate broom grass as a viable feedstock for bioethanol production, which is particularly important for rural economies in Thailand, where this species is prevalent.

Further research should focus on several key areas to optimize and scale up this process for industrial applications. Priority should be given to investigating high solid loadings (>15% *w*/*v*) during enzymatic hydrolysis to improve sugar concentrations and consequent ethanol yields, which is crucial for economic viability at industrial scale. Parallel efforts should be made to evaluate engineered strains of microorganisms with enhanced tolerance to pretreatment-derived inhibitors to maximize the fermentation performance and reduce the production costs. For industrial implementation, studies on continuous pretreatment processes and alkali recovery systems are essential, accompanied by a comprehensive techno-economic analysis to assess commercial viability, including:

− Production costs at various scales.
− Market value assessment of the main products and by-products.
− Waste management costs and strategies.
− Potential revenue streams from lignin and hemicellulose utilization.

Additionally, research on seasonal variations in biomass composition and their effects on pretreatment efficiency may be beneficial for establishing quality standards for broom grass as a reliable bioethanol feedstock. Finally, a detailed life-cycle analysis can yield crucial insights into both the environmental and economic sustainability of industrial implementation of this promising bioethanol production process.

## Figures and Tables

**Table 1:** Chemical composition (% dry weight) and crystallinity index of broom grass after NaOH-autoclave pretreatment with varying concentrations.

Composition	NaOH				
	Untreated	1%	2%	3%	4%
Glucan	32.9 ± 0.5 ^e^	48.3 ± 0.2 ^d^	54.6 ± 0.2 ^c^	57.3 ± 0.3 ^b^	61.3 ± 0.3 ^a^
Xylan	23.8 ± 0.2 ^a^	19.8 ± 0.3 ^b^	19.5 ± 0.1 ^b^	18.2 ± 0.3 ^c^	15.4 ± 0.2 ^d^
Arabinan	5.7 ± 0.0 ^a^	4.9 ± 0.2 ^b^	4.6 ± 0.1 ^c^	4.3 ± 0.2 ^d^	3.8 ± 0.0 ^e^
AIL	22.2 ± 0.5 ^a^	13.3 ± 0.4 ^b^	10.1 ± 0.1 ^c^	9.2 ± 0.2 ^d^	8.6 ± 0.1 ^e^
ASL	5.9 ± 0.1 ^a^	2.8 ± 0.1 ^b^	2.6 ± 0.1 ^c^	2.4 ± 0.0 ^d^	2.4 ± 0.0 ^d^
Total lignin	28.2 ± 0.4 ^a^	16.1 ± 0.4 ^b^	12.7 ± 0.1 ^c^	11.6 ± 0.2 ^d^	10.9 ± 0.1 ^e^
CrI	54.1%	57.8%	60.9%	62.6%	65.1%
Solid recovery	100 ^a^	62.5 ± 0.5 ^b^	56.2 ± 0.4 ^c^	53.4 ± 0.1 ^d^	48.9 ± 0.5 ^e^
Glucan recovery	100 ^a^	91.8 ± 0.4 ^c^	93.2 ± 0.3 ^b^	93.1 ± 0.5 ^b^	91.1 ± 0.5 ^c^
Xylan recovery	100 ^a^	52.0 ± 0.7 ^b^	46.0 ± 0.3 ^c^	40.7 ± 0.6 ^d^	31.7 ± 0.5 ^e^
Arabinan recovery	100	53.9 ± 2.3 ^b^	45.4 ± 1.0 ^c^	40.1 ± 2.2 ^d^	32.1 ± 0.3 ^e^
AIL recovery	100 ^a^	37.5 ± 1.1 ^b^	25.5 ± 0.3 ^c^	22.1 ± 0.4 ^d^	18.9 ± 0.2 ^e^
ASL recovery	100 ^a^	29.6 ± 0.7 ^b^	24.4 ± 0.7 ^c^	21.6 ± 0.2 ^d^	19.4 ± 0.3 ^e^
Total lignin recovery	100 ^a^	35.8 ± 1.0 ^b^	25.3 ± 0.1 ^c^	22.0 ± 0.3 ^d^	19.0 ± 0.1 ^e^
Lignin removal	0.0	64.2 ± 1.0 ^d^	74.7 ± 0.1 ^c^	78.0 ± 0.3 ^b^	81.0 ± 0.1 ^a^

The superscripted letters (a, b, c, d, and e) within rows indicate significant differences (*p* < 0.05). AIL: acid-insoluble lignin; ASL: acid-soluble lignin; CrI: crystallinity index.

**Table 2:** Chemical composition (% dry weight) and crystallinity index of broom grass after 2% NaOH-autoclave pretreatment at varying temperatures.

Composition (%Dw)	Temperature			
	Untreated	110 °C	120 °C	130 °C
Glucan	32.9 ± 0.5 ^d^	49.7 ± 0.6 ^c^	54.6 ± 0.2 ^b^	57.1 ± 0.2 ^a^
Xylan	23.8 ± 0.2 ^a^	20.8 ± 0.4 ^b^	19.5 ± 0.1 ^c^	18.7 ± 0.1 ^c^
Arabinan	5.7 ± 0.0 ^a^	4.8 ± 0.1 ^b^	4.6 ± 0.1 ^c^	4.5 ± 0.0 ^c^
AIL	22.2 ± 0.5 ^a^	14.9 ± 0.3 ^b^	10.1 ± 0.1 ^c^	8.5 ± 0.4 ^d^
ASL	5.9 ± 0.1 ^a^	2.9 ± 0.1 ^b^	2.6 ± 0.1 ^c^	2.4 ± 0.1 ^c^
Total lignin	28.2 ± 0.4 ^a^	17.9 ± 0.3 ^b^	12.7 ± 0.1 ^c^	10.9 ± 0.4 ^d^
CrI	54.1%	60.7%	60.9%	62.2%
Solid recovery	100 ^a^	58.2 ± 0.5 ^b^	56.2 ± 0.4 ^c^	54.2 ± 0.6 ^d^
Glucan recovery	100 ^a^	87.9 ± 1.0 ^c^	93.2 ± 0.4 ^b^	94.0 ± 0.2 ^b^
Xylan recovery	100 ^a^	50.8 ± 0.9 ^b^	46.0 ± 0.3 ^c^	42.6 ± 0.2 ^d^
Arabinan recovery	100 ^a^	48.4 ± 0.7 ^b^	45.5 ± 1.0 ^c^	42.7 ± 0.3 ^d^
AIL recovery	100 ^a^	29.1 ± 0.7 ^b^	25.5 ± 0.3 ^c^	20.7 ± 1.1 ^d^
ASL recovery	100 ^a^	28.8 ± 0.9 ^b^	24.4 ± 0.7 ^c^	21.9 ± 0.6 ^d^
Total lignin recovery	100 ^a^	36.9 ± 0.6 ^b^	25.3 ± 0.1 ^c^	20.9 ± 0.8 ^d^
Lignin removal	0.0 ^d^	63.1 ± 0.3 ^c^	74.7 ± 0.1 ^b^	79.1 ± 0.8 ^a^

The superscripted letters (a, b, c, d) within rows indicate significant differences (*p* < 0.05). AIL: acid-insoluble lignin; ASL: acid-soluble lignin; CrI: crystallinity index.

**Table 3:** Comparative assessment of alkaline pretreatment on previously investigated lignocellulosic substrates.

Feedstocks	Pretreatment Conditions	Lignin Removal (%)	Glucose Recovery (%)	Reference
*Chloris barbata*	2% NaOH, 110 °C, 60 min	71%	30.7%	[13]
Durian peel (Cultivar Monthong)	2% NaOH, 110 °C, 60 min	77%	36.1%	[31]
Durian peel (Cultivar Chanee)	2% NaOH, 110 °C, 60 min	74%	34.9%	[31]
*Sicyos angulatus*	2% NaOH, 110 °C, 10 min	-	8.5%	[32]
*Vietnamosasa pusilla*	2% NaOH, 120 °C, 60 min	50.5%	42.4%	[33]
Broom grass	2% NaOH, 120 °C, 60 min	74.7%	33.3%	This study

## Data Availability

The original contributions presented in the study are included in the article, further inquiries can be directed to the corresponding authors.

## Abbreviations

The following abbreviations are used in this manuscript:

AIL	Acid-insoluble lignin
ASL	Acid-soluble lignin
BHM	Biomass hydrolysate medium
CrI	Crystallinity index
DMRT	Duncan’s multiple-range test
GR	Glucose recovery
HEG	Hydrolysis efficiency of glucose
HPLC	High-performance liquid chromatography system
SEM	Scanning electron microscopy
SGM	Synthetic glucose medium
UV	Ultraviolet
XRD	X-ray diffraction
OD	Optical density

## References

[1] Abolore R.S., Jaiswal S., Jaiswal A.K. (2024). Green and sustainable pretreatment methods for cellulose extraction from lignocellulosic biomass and its applications: A review. Carbohydr. Polym. Technol. Appl..

[2] Khattab S.M.R., Okano H., Kimura C., Fujita T., Watanabe T. (2023). Efficient integrated production of bioethanol and antiviral glycerolysis lignin from sugarcane trash. Biotechnol. Biofuels Bioprod..

[3] Sharma S., Tsai M.-L., Sharma V., Sun P.-P., Nargotra P., Bajaj B.K., Chen C.-W., Dong C.-D. (2023). Environment friendly pretreatment approaches for the bioconversion of lignocellulosic biomass into biofuels and value-added products. Environments.

[4] Kaur S., Gaurav K. (2024). Lignocellulosic hydrolysates for the production of bioethanol: A comprehensive analysis. Sugar Tech..

[5] Johannes L.P., Xuan T.D. (2024). Comparative analysis of acidic and alkaline pretreatment techniques for bioethanol production from perennial grasses. Energies.

[6] Manandhar A., Sha A. (2018). Feedstock Logistics for Agricultural Residues and Energy Crops: Moving Biomass from the Field to Biorefinery Gate. Agriculture and Natural Resources. https://ohioline.osu.edu/factsheet/fabe-6604.

[7] Cheah W.Y., Sankaran R., Show P.L., Ibrahim T.N.B.T., Chew K.W., Culaba A., Chang J.-S. (2020). Pretreatment methods for lignocellulosic biofuels production: Current advances, challenges and future prospects. Biofuel Res. J..

[8] Zhu J.Y., Pan X., Zalesny R.S. (2010). Pretreatment of woody biomass for biofuel production: Energy efficiency, technologies, and recalcitrance. Appl. Microbiol. Biotechnol..

[9] El Hage M., Louka N., Rezzoug S.-A., Maugard T., Sablé S., Koubaa M., Debs E., Maache-Rezzoug Z. (2023). Bioethanol Production from Woody biomass: Recent advances on the effect of pretreatments on the bioconversion process and energy yield aspects. Energies.

[10] Bušić A., Marđetko N., Kundas S., Morzak G., Belskaya H., Ivančić Šantek M., Komes D., Novak S., Šantek B. (2018). Bioethanol production from renewable raw materials and its separation and purification: A review. Food Technol. Biotechnol..

[11] Mohapatra S., Mishra S.S., Bhalla P., Thatoi H. (2019). Engineering grass biomass for sustainable and enhanced bioethanol production. Planta.

[12] Premjet S., Doungporn P., Yoo H.Y., Kim S.W. (2018). Improvement of sugar recovery from *Sida acuta* (Thailand Weed) by NaOH pretreatment and application to bioethanol production. Korean J. Chem. Eng..

[13] Obeng A.K., Premjet D., Premjet S. (2019). Combining autoclaving with mild alkaline solution as a pretreatment technique to enhance glucose recovery from the invasive weed *Chloris barbata*. Biomolecules.

[14] Wongleang S., Premjet D., Premjet S. (2023). Cellulosic ethanol production from weed biomass hydrolysate of *Vietnamosasa pusilla*. Polymers.

[15] Kumar B., Bhardwaj N., Agrawal K., Chaturvedi V., Verma P. (2020). Current perspective on pretreatment technologies using lignocellulosic biomass: An emerging biorefinery concept. Fuel Process. Technol..

[16] Wongleang S., Premjet D., Premjet S. (2024). investigating the potential of grass biomass (*Thysanolaena latifolia*) as an alternative feedstock for sugar platforms and bioethanol production. Energies.

[17] Tiwari B.K., Shukla R.P., Lynser M.B., Tynsong H. (2012). Growth pattern, production, and marketing of *Thysanolaena maxima* (Roxb.) Kuntze: An important non-timber forest product of Meghalaya, India. For. Trees Livelihoods.

[18] Shukla A., Kumar D., Girdhar M., Kumar A., Goyal A., Malik T., Mohan A. (2023). Strategies of pretreatment of feedstocks for optimized bioethanol production: Distinct and integrated approaches. Biotechnol. biofuels bioprod..

[19] Zhao L., Sun Z.F., Zhang C.C., Nan J., Ren N.Q., Lee D.J., Chen C. (2022). Advances in pretreatment of lignocellulosic biomass for bioenergy production: Challenges and perspectives. Bioresour. Technol..

[20] Hute T., Sanusi I.A., Kana E.B.G., Meyer E.L., Sewsynker-Sukai Y. (2024). Comparative assessment of autoclave- and microwave-facilitated seawater pretreatments for the enhancement of sugar recovery from banana pseudostem. Biomass Convers. Biorefin..

[21] Valles A., Capilla M., Álvarez-Hornos F.J., García-Puchol M., San-Valero P., Gabaldón C. (2021). Optimization of alkali pretreatment to enhance rice straw conversion to butanol. Biomass Bioenergy.

[22] Haldar D., Purkait M.K. (2022). Thermochemical pretreatment enhanced bioconversion of elephant grass (*Pennisetum purpureum*): Insight on the production of sugars and lignin. Biomass Convers. Biorefin..

[23] Sawisit A., Jampatesh S., Jantama S.S., Jantama K. (2018). Optimization of sodium hydroxide pretreatment and enzyme loading for efficient hydrolysis of rice straw to improve succinate production by metabolically engineered *Escherichia coli* KJ122 under simultaneous saccharification and fermentation. Bioresour. Technol..

[24] Papathoti N.K., Laemchiab K., Megavath V.S., Keshav P.K., Numparditsub P., Le Thanh T., Buensanteai N. (2021). Augmented ethanol production from alkali-assisted hydrothermal pretreated cassava peel waste. Energy Source Part A Recovery Util. Environ. Eff..

[25] Kontogianni N., Barampouti E.M., Mai S., Malamis D., Loizidou M. (2019). Effect of alkaline pretreatments on the enzymatic hydrolysis of wheat straw. Environ. Sci. Pollut. Res..

[26] Sluiter A., Hames B., Ruiz R., Scarlata C., Sluiter J., Templeton D., Crocker D. (2012). Determination of Structural Carbohydrates and Lignin in Biomass.

[27] Premjet S., Dana S., Obeng A.K., Premjet D. (2018). Enzymatic response to structural and chemical transformations in *Hibiscus sabdariffa* var. altissima bark and core during phosphoric acid pretreatment. BioResources.

[28] Segal L., Creely J.J., Martin Jr A., Conrad C. (1959). An empirical method for estimating the degree of crystallinity of native cellulose using the X-ray diffractometer. Text. Res. J..

[29] Saratale G.D., Oh M.-K. (2015). Improving alkaline pretreatment method for preparation of whole rice waste biomass feedstock and bioethanol production. RSC Adv..

[30] Bala R., Mondal M.K. (2018). Exhaustive characterization on chemical and thermal treatment of sawdust for improved biogas production. Biomass Convers. Biorefin..

[31] Obeng A.K., Premjet D., Premjet S. (2021). Improved glucose recovery from durian peel by alkaline-catalyzed steam pretreatment. PeerJ.

[32] An H.-E., Lee K.H., Jang Y.W., Kim C.-B., Yoo H.Y. (2021). improved glucose recovery from *Sicyos angulatus* by NaOH pretreatment and application to bioethanol production. Processes.

[33] Wongleang S., Premjet D., Premjet S. (2023). Physicochemical pretreatment of *Vietnamosasa pusilla* for bioethanol and xylitol production. Polymers.

[34] Wang S., Li F., Zhang P., Jin S., Tao X., Tang X., Ye J., Nabi M., Wang H. (2017). Ultrasound assisted alkaline pretreatment to enhance enzymatic saccharification of grass clipping. Energy Convers. Manag..

[35] Xu J., Cheng J.J., Sharma-Shivappa R.R., Burns J.C. (2010). Sodium hydroxide pretreatment of switchgrass for ethanol production. Energy Fuels.

[36] Wunna K., Nakasaki K., Auresenia J.L., Abella L.C., Gaspillo P.-a.D. (2017). Effect of alkali pretreatment on removal of lignin from sugarcane bagasse. Chem. Eng. Trans..

[37] Shah T.A., Khalid S., Nafidi H.-A., Salamatullah A.M., Bourhia M. (2023). Sodium hydroxide hydrothermal extraction of lignin from rice straw residue and fermentation to biomethane. Sustainability.

[38] Modenbach A.A., Nokes S. (2014). Effects of sodium hydroxide pretreatment on structural components of biomass. Trans. ASABE.

[39] Nath P., Maibam P.D., Singh S., Rajulapati V., Goyal A. (2021). Sequential pretreatment of sugarcane bagasse by alkali and organosolv for improved delignification and cellulose saccharification by chimera and cellobiohydrolase for bioethanol production. 3 Biotech.

[40] Matsushita Y., Kakehi A., Miyawaki S., Yasuda S. (2004). Formation and chemical structures of acid-soluble lignin II: Reaction of aromatic nuclei model compounds with xylan in the presence of a counterpart for condensation, and behavior of lignin model compounds with guaiacyl and syringyl nuclei in 72% sulfuric acid. J. Wood Sci..

[41] Xu H., Li B., Mu X. (2016). review of alkali-based pretreatment to enhance enzymatic saccharification for lignocellulosic biomass conversion. Ind. Eng. Chem. Res..

[42] Hoşgün E.Z., Bozan B. (2020). Effect of different types of thermochemical pretreatment on the enzymatic hydrolysis and the composition of *Hazelnut Shells*. Waste Biomass Valori..

[43] Li C., Fan M., Xie J., Zhang H. (2023). Effect of NaOH-catalyzed organosolv pretreatment on the co-production of ethanol and xylose from poplar. Ind. Crops. Prod..

[44] Sun S., Sun S., Cao X., Sun R. (2016). The role of pretreatment in improving the enzymatic hydrolysis of lignocellulosic materials. Bioresour. Technol..

[45] Ishiguro M., Endo T. (2014). Addition of alkali to the hydrothermal–mechanochemical treatment of Eucalyptus enhances its enzymatic saccharification. Bioresour. Technol..

[46] Gomes C.L., Gonçalves E., Suarez C.A.G., de Sousa Rodrigues D., Montano I.C. (2021). Effect of reaction time and sodium hydroxide concentration on delignification and enzymatic hydrolysis of brewer’s spent grain from two brazilian brewers. Cellulose Chem. Technol..

[47] Satari B., Karimi K., Kumar R. (2019). Cellulose solvent-based pretreatment for enhanced second-generation biofuel production: A review. Sustain. Energy Fuels.

[48] Kumar A., Singh S., Rajulapati V., Goyal A. (2020). Evaluation of pre-treatment methods for Lantana camara stem for enhanced enzymatic saccharification. 3 Biotech.

[49] Debiagi F., Madeira T.B., Nixdorf S.L., Mali S. (2020). Pretreatment efficiency using autoclave high-pressure steam and ultrasonication in sugar production from liquid hydrolysates and access to the residual solid fractions of wheat bran and oat hulls. Appl. Biochem. Biotechnol..

[50] Gao F., Wang H., Li F., De Y., Ma Y., Jiang H., Jing Y., Qu H., Li P. (2022). Evaluation of lignocellulose degradation and ethanol production via dilute acid and alkali pretreatment of hybrid Pennisetum. BioRes..

[51] Xu F., Shi Y.-C., Wang D. (2013). X-ray scattering studies of lignocellulosic biomass: A review. Carbohydr. Polym..

[52] Wang X., Le H., Guo Y., Zhao Y., Deng X., Zhang J., Zhang L. (2022). Preparation of cellulose nanocrystals from jujube cores by fractional purification. Molecules.

[53] Zhang Z., Zheng H., Qian J. (2023). Pretreatment with a combination of steam explosion and NaOH increases butanol production of enzymatically hydrolyzed corn stover. Renew. Energy.

[54] Manokhoon P., Rangseesuriyachai T. (2020). Effect of two-stage sodium hydroxide pretreatment on the composition and structure of Napier grass (Pakchong 1) (*Pennisetum purpureum*). Int. J. Green Energy.

[55] Wang Z., Wu S., Fan C., Zheng X., Zhang W., Wu D., Wang X., Kong H. (2021). Optimisation of enzymatic saccharification of wheat straw pre-treated with sodium hydroxide. Sci. Rep..

[56] Wu W., Li P., Huang L., Wei Y., Li J., Zhang L., Jin Y. (2023). The Role of Lignin Structure on Cellulase Adsorption and Enzymatic Hydrolysis. Biomass.

[57] Junior C.S., Milagres A.M.F., Ferraz A., Carvalho W. (2013). The effects of lignin removal and drying on the porosity and enzymatic hydrolysis of sugarcane bagasse. Cellulose.

[58] Li M., Zhang Q., Chen C., Wang S., Min D. (2019). Lignin interaction with cellulase during enzymatic hydrolysis. Pap. Biomater..

[59] Cui P., Ye Z., Chai M., Yuan J., Xiong Y., Yang H., Yao L. (2022). Effective fractionation of lignocellulose components and lignin valorization by combination of deep eutectic solvent with ethanol. Front. Bioeng. Biotechnol..

[60] Zoghlami A., Paës G. (2019). Lignocellulosic biomass: Understanding recalcitrance and predicting hydrolysis. Front. Chem..

[61] Lee J., Kim S., Lee K.H., Lee S.K., Chun Y., Kim S.W., Park C., Yoo H.Y. (2022). Improvement of bioethanol production from waste chestnut shells via evaluation of mass balance-based pretreatment and glucose recovery process. Environ. Technol. Innov..

[62] Zhao X., Huang C., Lin W., Bian B., Lai C., Ling Z., Yong Q. (2022). A structure–activity understanding of the interaction between lignin and various cellulase domains. Bioresour. Technol..

[63] Zhang Q., Wan G., Li M., Jiang H., Wang S., Min D. (2020). Impact of bagasse lignin-carbohydrate complexes structural changes on cellulase adsorption behavior. Int. J. Biol. Macromol..

[64] Moysés D.N., Reis V.C., Almeida J.R., Moraes L.M., Torres F.A. (2016). Xylose fermentation by *Saccharomyces cerevisiae*: Challenges and prospects. Int. J. Mol. Sci..

[65] Endalur Gopinarayanan V., Nair N.U. Pentose metabolism in *Saccharomyces cerevisiae*: The need to engineer global regulatory systems. Biotechnol. J..

[66] Topaloğlu A., Esen Ö., Turanlı-Yıldız B., Arslan M., Çakar Z.P. (2023). From *Saccharomyces cerevisiae* to ethanol: Unlocking the power of evolutionary engineering in metabolic engineering applications. J. Fungi.

[67] Jönsson L.J., Martín C. (2016). Pretreatment of lignocellulose: Formation of inhibitory by-products and strategies for minimizing their effects. Bioresour. Technol..

[68] Kim J.S., Lee Y.Y., Kim T.H. (2016). A review on alkaline pretreatment technology for bioconversion of lignocellulosic biomass. Bioresour. Technol..

[69] Zhang J., Geng A., Yao C., Lu Y., Li Q. (2012). Effects of lignin-derived phenolic compounds on xylitol production and key enzyme activities by a xylose utilizing yeast *Candida athensensis* SB18. Bioresour. Technol..

[70] Rage G., Atasi O., Wilhelmus M.M., Hernández-Sánchez J.F., Haut B., Scheid B., Legendre D., Zenit R. (2020). Bubbles determine the amount of alcohol in Mezcal. Sci. Rep..

[71] Lin Y., Zhang W., Li C., Sakakibara K., Tanaka S., Kong H. (2012). Factors affecting ethanol fermentation using *Saccharomyces cerevisiae* BY4742. Biomass Bioenergy.

[72] Stanley D., Fraser S., Chambers P.J., Rogers P., Stanley G.A. (2010). Generation and characterisation of stable ethanol-tolerant mutants of *Saccharomyces cerevisiae*. J. Ind. Microbiol. Biotechnol..

